# Work-related leukemia: a systematic review

**DOI:** 10.1186/1745-6673-8-14

**Published:** 2013-05-22

**Authors:** Ioannis Polychronakis, George Dounias, Vasilios Makropoulos, Elena Riza, Athena Linos

**Affiliations:** 1 Department of Hygiene, Epidemiology and Medical Statistics, University of Athens, Medical School, 75 Mikras Asias 115 27, Goudi, Athens, Greece; 2 Occupational & Industrial Hygiene Department, National School of Public Health, 96 Alexandras Av. 11521, Athens, Greece

**Keywords:** Leukemia, Exposure, Occupation, Worker, Work-related, Hazard, Risk-factor

## Abstract

Leukemia is a complex disease, which only became better understood during the last decades following the development of new laboratory techniques and diagnostic methods. Despite our improved understanding of the physiology of the disease, little is yet known about the causes of leukemia. A variety of potential risk factors have been suggested so far, including personal habits and lifestyle, and a wide range of occupational or environmental exposures. A causal association with leukemia has only been documented to date for ionizing radiation, benzene and treatment with cytostatic drugs, but there is an ongoing scientific debate on the possible association of leukemia with a number of other work-related hazards. In this article, we have reviewed scientific studies, published over the past 5 years, which investigated potential associations between leukemia and exposure to occupational risk factors. The systematic literature review took place via electronic databases, using specific search criteria, and independent reviewers have further filtered the search results to identify the number of articles, presented in our paper. A large number of studies included in the review referred to the effects of ionizing radiation, where new data suggest that the effects of exposure to small doses of ionizing radiation should probably be reevaluated. Some other works appear to substantiate a potential association of the disease with certain pesticides. Further research is also suggested regarding the role of infectious agents or exposure to certain chemicals like formaldehyde or butadiene in the pathogenesis of leukemia.

## Review

### Introduction

The term "leukemia" refers to a group of diseases with different biological background, clinical presentation, prognosis and response to treatment, characterized by a malignant transformation of hematopoietic cells which produce an abnormal leukemic population (clone) of cells suppressing the production of normal blood cellular components. The disease was first identified in the mid-19th century when different researchers described a common pathology caused by abnormalities of the white blood cells [1], hence the term “Leukemia” which originates from the Greek words “Leuko” (white) and “Haema” (blood). While scientific research has advanced significantly since then as regards to the underlying pathophysiology of the various types of the disease, our knowledge on the causative factors involved in the development of leukemia is still limited.

Different researchers have proposed a number of potential risk factors for leukemia, but to date exposure to ionizing radiation, alkylating agents and benzene [2] remain the only hazards for which an association with leukemia has been substantiated.

An association between ionizing radiation and leukemia was first assumed in the early 20th century, following the effects of uncontrolled exposure of patients and medical personnel to the radiation used for diagnostic and therapeutic purposes [1,3]. Those observations were further corroborated by the findings of Life Span Study (LSS) among the A-Bomb survivors of Hiroshima and Nagasaki [4-7], which persist for decades following exposure to radiation [8,9] and the follow-up studies among cleaning-workers in Chernobyl reactor site [10].

Benzene is according to the current scientific knowledge the second best-documented risk factor for the development of leukemia [11]. Its chemical properties have led to its widespread use as a solvent in a number of industrial applications, and occupational exposure to benzene has been documented for workers in different production sectors i.e. production of chemicals, pharmaceuticals, plastics, synthetic rubbers, paints, oil processing etc. [12-14]. Epidemiological studies have shown a significant association between exposure to Benzene and the incidence of acute myeloid leukemia (AML) [15-17].

Cytostatic drugs, and especially chemotherapeutic regimens containing alkylating agents such as Busulfan, Chlorambucil, Cyclophosphamide etc. used in the treatment of solid organ malignancies are another documented risk factor for developing leukemia, as a number of clinical trials on cancer therapeutics has shown an association between this category of drugs and the development of secondary leukemia [18], particularly AML [19]. Despite their known toxicity to oncology patients and the precautionary measures taken to prevent occupational exposure, a number of workers from different disciplines could be exposed to significant concentrations of cytostatic drugs during their production, transportation, distribution, preparation and administration to patients [20-22]. Nevertheless, little is known regarding the potential effect of this exposure on the risk of developing the disease [23-26].

Taking into account the limited knowledge on its actual causes, the investigation of the role of different work-related risk factors in the development of leukemia would be of great scientific interest, providing valuable insight on the etiology of the disease and the available options for the protection of health of specific categories of workers or the public, given the widespread use of different chemical compounds and technological applications in common consumer products.

The growing number of related publications over the last years indicates an increasing concern of the scientific community and recent studies have presented new information as regards our cumulative experience from the application of different technologies and chemical or biological factors in industry, necessitating a review of the current literature on the subject.

Therefore through this paper we systematically collected and summarized all relevant scientific information that has been published internationally in the English language over the last years, exploring the potential role of occupational "risk factors" in the development of the disease.

### Materials and methods

The article review on the role of occupational risk factors in the development of leukemia took place between September and December 2010. The literature search was carried out via the internet, using the online medical database "Pubmed" supported by the US National Library of Medicine and the more generic search-engine "Google Scholar".

**Figure 1:**
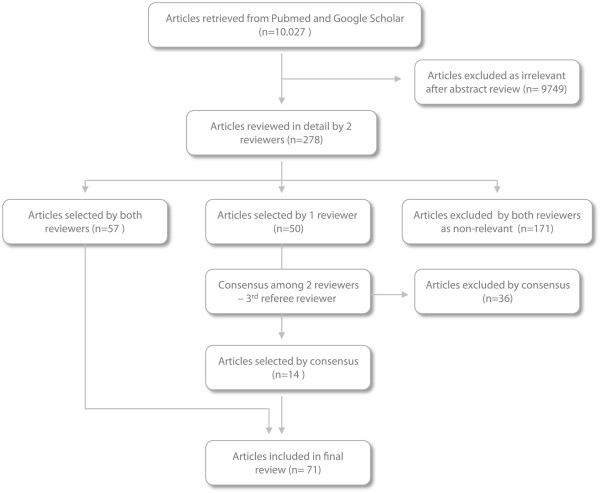
**Article selection process for the review.** The chart presented in this figure shows the overall process for the selection of articles from the initial electronic search to the final selection of the reviewed studies.

The methodology followed by the research team for the selection of articles is presented schematically in Figure 1.

At a first stage, all published articles containing the word "leukemia” as part of their title, as a key-word or as a reference in their full text have been collected.

A set of criteria was adopted to narrow down the search results, excluding studies published more than 5 years prior to the review, studies that referred to subjects younger than 18 years (since the review referred to work-related risk factors) and studies whose full text was not available in English. All types of publications (i.e. prospective or retrospective studies, original articles, literature reviews, meta-analyses etc.) were considered at this stage.

The initial search recovered more than 10.000 articles (629 literature reviews) who were filtered by 2 different reviewers as regards their pertinence to the scopes of the study. The reviewers screened the abstracts of all collected articles and considered for further review only studies that referred to the potential effect of various risk factors (exposures) in the development of leukemia. The occupational or environmental nature of exposure was not examined at this stage. The review investigated information on the potential role of occupational risk factors regardless of the research type (toxicological, molecular, epidemiological study etc.) therefore no selection took place on the basis of specific study design. Nevertheless, with the exception of a number of articles who were further reviewed to elucidate potential points of interest, the overwhelming majority of clinical trials and laboratory studies were clearly non-relevant to the context of the review and were directly excluded, limiting the number of articles at the end of second phase to 278.

For the final selection process the remaining articles were examined in detail by both reviewers. In cases of agreement, the corresponding articles were either excluded or included in the final list, while in cases of ambiguity there was further discussion among the 2 reviewers and a third reviewer who played the role of a referee, in order to reach consensus.

Articles considered eligible for inclusion were those that referred to specific categories of workers or exposure to occupational hazards and their effect on leukemia. The role of environmental exposures on leukemia was beyond the scope of this paper and all related studies were subsequently excluded.

A limited number of the reviewed articles on ionizing radiation and benzene did not refer specifically to occupational exposure, but their findings were considered significant for understanding the role of those hazards in occupational settings and were thus included in the final list. Finally, while leukemia in children was beyond the objectives of this study, the effect of parental occupational exposure on the risk of the disease among their offspring was considered work-related and therefore similar studies were also included in the final review.

The 71 final studies, along with their main findings are presented in Tables 1, 2 and 3 classified according to the author, year, type of study and type of exposure. Table 1 presents the reviewed articles on physical hazards, Table 2 presents articles on chemical and biological hazards and Table 3 refers to others types of hazards and specific categories of workers.

**Table 1:** Reviewed articles on physical hazards and leukemia

**Researcher**	**Year**	**Type of study**	**Exposure**	**Main findings**
**A. Ionizing radiation and leukemia**				
**1) Update of LSS study findings**				
Little et al. [6]	2009	Review	Ionizing radiation	Leukemia RR follows the pattern of LSS study for low dose (occupational) exposure - Lower RR per dose unit for higher doses of radiation
Richardson et al. [9]	2009	Cohort study	Ionizing radiation	Exposure to >5 mGy of radiation is responsible for 1/3 of leukemia cases after 5 decades – ERR/Gy follows a quadratic dose-response model for AML. ALL and CML mortality follow a linear dose-response model.
**2) Cleanup workers employed in Chernobyl nuclear incident**				
Rahu et al. [27]	2006	Cohort study	Ionizing radiation	No significant increase in leukemia incidence among workers (SIR 1.53, 95% CI 0.62 - 3.17). A marginally significant increase has been observed among Latvian workers but it was based on a small number of cases.
Abramenko et al. [28]	2008	Cohort study	Ionizing radiation	Among a cohort of CLL patients, specific genetic polymorphisms where observed more frequently among cleanup workers exposed to radiation following the nuclear accident in Chernobyl than in non-exposed CLL patients
Romanenko et al. [29,30]	2008	Nested case-control study	Ionizing radiation	Positive linear trend (p = 0.03) between increasing exposure to radiation and leukemia risk. The ERR/Gy for leukemia was 3.44 (95% CI 0.47 – 9.78). A linear dose - response relationship has been shown for ALL and (surprisingly) for CLL.
Kesminiene et al. [31]	2008	Case-control study	Ionizing radiation	A statistically significant association was shown (at 90% but not at 95% level) between AL and employment as a cleanup worker in the surrounding area (<30 km) of Chernobyl accident site (OR 8.31, 90% CI 1.17 - 122).
**3) Workers in nuclear industry**				
Boice et al. [32]	2006	Cohort study	Ionizing radiation	No significant increase in leukemia mortality (SMR 1.21, 95% CI 0.69- 1.97) or increased leukemia risk for the highly-exposed group (RR 1.34, 95% CI 0.73–2.45) was shown among workers.
Richardson et al. [33]	2007	Cohort study	Ionizing radiation (& exposure to chemicals)	A borderline significant increase of leukemia mortality was shown (at 90% but not at 95% level) only for operators and manual workers (SMR 1.36, 90% CI 1.02 - 1.78) and for workers employed >30 years (SMR 1.63, 90% CI 1.07 - 2.52).
Richardson et al. [34]	2007	Nested case-control study	Ionizing radiation	Assuming a 3-year time lag, no significantly increased ERR/10 mSv was shown for all leukemias (0.041, 90% CI -0.001 – 0.116), for leukemias excluding CLL (0.077, 90% CI 0.014 – 0.198) or for myeloid leukemia (0.123, 90% CI 0.021 - 0.354).
Schubauer-Berigan et al. [35]	2007	Nested case-control study	Ionizing radiation	A non-significant positive association between radiation dose and leukemia risk was shown for doses 10 - 100 mSv, with an estimated ERR/10 mSv of 0.068 (95% CI -0.029 - 0.24).
Schubauer-Berigan et al. [36]	2007	Nested case-control study	Ionizing radiation	A non-significant positive association between radiation dose and CLL risk was shown for doses 10 - 100 mSv, with an estimated ERR/10 mSv of 0.20 (95% CI -0.035 – 0.96).
Matanoski et al. [37]	2008	Cohort study	Ionizing radiation	No statistically significant increase of leukemia mortality (SMR 0.91, 95% CI 0.56 – 1.39 and 0.42, 95% 0.11 – 1.07 for exposure to > 5.0 mSv and <5.0 mSv of radiation respectively) was shown among workers
Ashmore et al. [38]	2010	Review	Ionizing radiation	Previous study of IARC (2005) who found no statistically significant association between leukemia and radiation exposure among workers in nuclear industry could be biased due to inaccurate estimation of exposure.
**4) Medical applications of radiation**				
Lie et al. [39]	2008	Cohort study	Ionizing radiation	No significant increase in leukemia risk was found for the group nurses with the longest (> 30 years) employment in posts exposed to radiation compared to the group of non-exposed nurses (RR 0.77, 95% CI 0.35 – 1.69).
Samerdokiene et al. [40]	2009	Cohort study	Ionizing radiation	No significant increase in leukemia incidence (SIR 3.3, 95% CI 0.68- 9.63 for men and 2.67, 95% CI 0.92-4.2 for women) was shown among personnel employed in medical applications of ionizing radiation.
Ramos et al. [41]	2008	Case-control study (exposure assessment)	Ionizing radiation	The projected risk of leukemia cases/1000 person-years based on cumulative radiation exposure among a group of interventional radiologists differed between 2 methods of exposure assessment (1.07-3.98 according to the physical method compared to 1.07-11.21 for the biological method) suggesting a potential improper use of personal dosimeters.
Ramos et al. [42]	2009	Molecular epidemiological study	Ionizing radiation	The projected LAR of leukemia (cases/1000 person-years) among interventional radiologists due to radiation exposure, was much higher according to biological methods of exposure assessment (9.2) compared to physical methods (2.18).
**5) Industrial applications of ionizing radiation**				
Ahn et al. [43]	2008	Cohort study	Ionizing radiation (industrial applications)	No statistically significant increase of leukemia SMR or SRR was shown among personnel exposed to radiation (workers in medical applications, research laboratories, nuclear facilities, non-destructive testing, military facilities etc.).
**6) Extraction and use of uranium compounds**				
Storm [44]	2006	Cohort study	Depleted uranium	No statistically significant increase of leukemia incidence was found among military personnel exposed to depleted uranium used during military operations (SIR 1.4, 95% CI 0.4 - 3.5).
Mohner [45]	2006	Case-control study	Uranium mining (radionuclides)	No significant increase of leukemia risk was found in the group with the highest cumulative exposure to radon (> 400 mSv) compared to the low exposure group (OR 2.21, 90% CI 1.25–3.91).
Mohner [46]	2010	Case-control study	Uranium mining (radionuclides)	No significant increase of leukemia risk was found among the group of workers with the highest (> 200 mSv) cumulative exposure (OR 1.33, 90% CI 0.82-2.14).
**B. Non-ionizing radiation (EMF) and leukemia**				
Roosli [47]	2007	Cohort-study	ELF EMF	A significantly increased Hazard Ratio was shown for myeloid leukemia among the workers with the highest exposure to ELF EMF (HR 4.74, 95% CI 1.04-21.6, p=0.035).
Kheifets [48]	2008	Meta-analysis	EMF	A small but statistically significant increase in leukemia risk was found among the exposed group (RR 1.16, 95% CI 1.11-1.22 for all leukemias).

**Table 2:** Reviewed articles on chemical and biological hazards and leukemia

**Researcher**	**Year**	**Type of study**	**Exposure**	**Main findings**
**1) Exposure to benzene**				
Zhang [49]	2007	Molecular epidemiological study	Exposure to benzene	A statistically significant increase % of genetic aberrations commonly observed in chemotherapy-related leukemias has been shown among workers exposed to benzene compared to non-exposed controls.
Richardson [50]	2009	Cohort study	Exposure to benzene	The observed pattern of leukemia mortality among rubber-production workers exposed to benzene, compared to the prediction of a TSCE statistical model suggests that benzene plays a role to the kinetics of cancer cells rather than the initial malignant transformation.
Rushton [51]	2010	Ecological study	Exposure to benzene	The Attributable Fraction (AF) of population mortality from acute non-lymphocytic leukemia related to occupational exposure to benzene was estimated at 0.25% (95% CI 0.0-4.65).
**2) Exposure to organic solvents (incl. benzene)**				
Costantini et al. [52]	2008	Polycentric case-control study	Organic solvents	A significant increase of CLL risk has been shown among the groups of workers with moderate – high exposure to Benzene (OR 1.8, 95% CI 0.9-3.9), and high exposure to Xylene (OR 1.9, 95% CI 0.8-4.5) and Toluene (OR 2.1, 95% CI 0.9-4.7).
Cocco et al. [53]	2010	Case-control study	Organic solvents	A small but statistically significant increase of CLL risk (OR 1.3, 95% CI 1.1-1.6) was shown for workers ever exposed to organic solvents (exposure to any solvent or combined exposure of benzene with toluene, xylene or gasoline) compared to non-exposed.
Lehman et al. [54]	2006	Cohort study	Organic solvents	No statistically significant increase in mortality from leukemia was found among exposed workers in footwear industry, compared with the general population (SMR 1.01, 95% CI 0.67-1.48).
**3) Exposure to Dioxins**				
Atallah et al. [55]	2007	Case-report	Dioxins – Agent Orange	A rare case of Acute Promyelocytic Leukemia (APL) in a former USAF pilot, who was involved in spraying Agent Orange during the Vietnam War, was reported.
Collins et al. [56]	2009	Cohort-study	TCDD	No significant increase of leukemia mortality was shown among exposed workers (SMR 1.9, 95% CI 1.0- 3.2). No significant association was shown between leukemia mortality and cumulative exposure (ppb) to TCDD (p=0.34).
**4) Exposure to chemical compounds used in the synthetic rubber industry**				
Delzell et al. [57]	2006	Cohort-study	BD, DMDTC and Styrene	A positive association was shown between (all) leukemias, CML and CLL mortality and occupational exposure to BD. A positive association was also found between leukemia and exposure to Styrene or DMDTC, but in both cases there was also co-exposure to BD.
Beall et al. [58]	2007	Cohort-study	Solvents, aromatic amines	No increased leukemia incidence (SIR 0.68, 95% CI 0.14-1.98) or mortality (SMR 0.95, 95% CI 0.31- 2.23) has been observed among exposed workers, compared to the general population.
Cheng et al. [59]	2007	Cohort-study	BD	A statistically significant association was shown between leukemia risk and exposure to BD, for cumulative exposure in ppm-years (RR 3.84, 95% CI 1.51- 9. 76), frequency of exposure (RR 4.26, 95% CI 1.62-11.21) and average exposure in ppm (RR 3.93, 95% CI 1.5-10.32).
Sathiakumar et al. [60,61]	2009	Cohort-study	BD and Styrene	In contrast to the results observed among men workers, no statistically significant association was found between exposure of women workers to BD and mortality from leukemia (SMR 0.78, 95% CI 0.38-1.44).
**5) Exposure to Formaldehyde**				
Beane Freeman [62]	2009	Cohort-study	Formaldehyde	A non-significant increase in mortality from (all) leukemias (RR 1.42, 95% CI 0.92- 2.18) and myeloid leukemia (RR 1.78, 95% 0.87- 3.64) was shown in the group with the highest exposure (> 4ppm) compared to the group with the lowest exposure.
Hauptmann [63]	2009	Case-control study	Formaldehyde	A statistically significant association between mortality from myeloid leukemia and increasing years of employment (p=0.02) or maximum exposure to formaldehyde (p=0.036) was shown among embalmers.
Zhang [64]	2010	Molecular epidemiological study	Formaldehyde	A statistically significant decrease of cell-lines, reduced activity of the CFU-GMs and increase of leukemia-related genetic aberrations has been shown among workers exposed to formaldehyde compared to non-exposed controls.
Speit [65]	2010	Letter to the editor	Formaldehyde	A number of methodological issues call into question the reliability of the findings of the study of Zhang et al, on in-vitro evidence of leukemia-specific chromosomal changes in workers exposed to formaldehyde.
**6) Exposure to Lead**				
Lam [66]	2007	Cohort study	Occupational exposure to lead	A non-significant increase of CML incidence was shown (SIR 1.75, 95% CI 0.02- 9.71) among the cohort of workers exposed to lead (metal constructions, metal processing industry, foundries, manufacture of batteries and electronics, glass production).
**7) Exposure to different types of pesticides, herbicides and insecticides**				
Mahajan [67]	2006	Cohort study - (AHS)	Organophosphate pesticide - Fonofos	A statistically significant increase of leukemia risk was found among pesticide applicators with the highest exposure (based on duration and intensity of exposure) compared to the non-exposed (RR 2.67, 95% CI 1.06-6.70).
Mahajan [68]	2006	Cohort study - (AHS)	Organophosphate pesticide - Phorate	The small number of recorded cases of leukemia among exposed workers did not allow for any reliable conclusions.
Miligi [69]	2006	Case-control study	Different groups of pesticides	No significant association with leukemia risk was shown for exposure to fungicides (OR 1.0, 95% CI 0.7 -1.3), herbicides (OR 1.4, 95% CI 0.8-2.3), insecticides (OR 1.0, 95% CI 0.7 -1.4), molluscicides (OR 0.9, 95% CI 0.3-2.5) or rodenticides (OR 0.4, 95% CI 0.1-1.2).
Hansen [70]	2007	Cohort study	Arsenate pesticides, Atrazine, Dichlorvos, Captafol, Amitrol, Lindane, DDT	A significant increase of leukemia incidence (SIR 2.33, 95% CI 1.32- 4.10) was found among the group of gardeners with high exposure to pesticides, previous to the 1960 restriction of the use of potentially carcinogenic substances.
Purdue [71]	2007	Cohort study - (AHS)	Organochlorine insecticides (Aldrin, Chlordane, DDT, Dieldrin, Heptachlor, Lindane, Toxaphene)	A marginal statistically significant association was found between the leukemia risk and previous use of any of the Organochlorine pesticides (RR 2.0, 95% CI 1.0-4.1), Lindane (RR 2.0, 95% CI 1.1-3.5) or Heptachlor (RR 2.1, 95% CI 1.1-3.9).
van Bemmel [72]	2008	Cohort study - (AHS)	Thio-carbamate herbicide EPTC	No statistically significant increase of leukemia risk was shown for workers exposed EPTC (RR 1.31, 95% CI 0.75-2.28) compared to the non-exposed.
Chrisman Jde [73]	2009	Ecological study	All pesticides	A statistically significant increase of leukemia mortality was observed in areas with increased per capita use of pesticides (MRR 1.6, 95% CI 1.55-1.66 and 1.93, 95% CI 1.87-2.0 for the 1^st^ quartile of pesticide use, compared to the 2^nd^ and 3^rd^ quartile respectively).
Delancey [74]	2009	Cohort study - (AHS)	Herbicide Metribuzin	A statistically non-significant increase of leukemia risk was shown among the group of pesticide applicators with the highest cumulative exposure to Metribuzin (RR 2.42, 95% CI 0.82-7.19, p=0.08).
Orsi [75]	2009	Case-control study	Organochlorine insecticides, Phenoxy – herbicides, Triazine-containing herbicides	A significant association was shown between the risk of hairy cell leukemia (HCL) and exposure to Organochlorine insecticides (OR 4.9, 95% CI 1.1-21.2), Phenoxy-herbicides (OR 4.1, 95% CI 1.1-15.5) and Triazine-containing herbicides (OR 5.1, 95% CI 1.4-19.3).
Rusiecki [76]	2009	Cohort study - (AHS)	Exposure to Permethrin	No significant association was shown between exposure to Permethrin and leukemia risk among workers (RR 1.74, 95% CI 0.83 - 3.64 and 1.34, 95% CI 0.61-2.92 for workers with the longest duration of exposure or the highest cumulative exposure respectively).
**8) Combined chemical and biological hazards in agriculture**				
Bassil [77]	2007	Literature review	Combined exposure to pesticides, insecticides and work with livestock	6 cohort studies and 8 case-control studies have shown a statistically significant association between pesticide exposure and leukemia risk, and 2 cohort studies have shown an association between leukemia risk and work with livestock.
**9) Biological hazards in agriculture and food industry**				
Moore [78]	2007	Case-control study (multicentric)	Occupational exposure to meat products	A statistically significant association was found between CLL risk and occupational exposure to meat products, for workers with exposure> 16 years to cattle and poultry meat (OR 2.51, 95% CI 1.12 - 5.66 and 2.06, 95% CI 1.17 - 3.63 respectively).
Johnson [79]	2010	Cohort study	Occupational exposure to meat products	A statistically significant increase of lymphatic leukemia mortality was shown among men workers in slaughterhouses and poultry meat processing plants (SMR 5.9, 95% CI 1.6-15.2). No similar increase was found among female workers.
Johnson [80]	2010	Cohort study	Occupational exposure to meat products	A statistically significant increase of lymphatic leukemia proportional mortality was observed only among non-white women workers in slaughterhouses and poultry meat processing plants (PMR 6.4, 95% CI 1.3-31.1).

**Table 3:** Reviewed articles on other hazards and leukemia

**Researcher**	**Year**	**Type of study**	**Exposure**	**Main findings**
**A) Occupational exposure and risk of leukemia among different categories of workers**				
**1) Healthcare workers**				
Dimich-Ward [81]	2007	Cohort study	Working as a nurse	No increased leukemia mortality (SMR 0.78, 95% CI 0.49-1.18) or incidence was found among the cohort of nurses (SIR for myelogenous and lymphatic leukemia was 1.21, 95% CI 0.85- 1.68 and 1.02, 95% CI 0.63-1.56 respectively).
Abel [82]	2009	Cohort study (Nurses’ Health Study)	Working as a nurse	A statistically significant increase of CLL incidence was found among nurses compared to the general population (SIR 1.35, 95% CI 1.17-1.54).
Lollis [83]	2010	Cohort study	Exposure to different health hazards of operating theaters	No statistically significant increase in mortality from leukemia was observed among neurosurgeons compared to the general population (SMR 1.2, 95% CI 0.75-1.9).
**2) Workers in chemical laboratories**				
Kubale [84]	2008	Cohort study	Radionuclides, benzene and other hazards in the laboratories of nuclear research facilities	Leukemia mortality of workers did not differ significantly from the general population (SMR 0.78, 95% CI 0.45-1.25). A significant positive association was shown between leukemia risk and duration of employment for those employed >20 years (SRR was 9.51, 95% CI 1.67-54.17 and 11.44, 95% CI 1.88-69.54, for an estimated 2- or 5-year time-lag of the disease respectively).
**3) Firefighters**				
Bates [85]	2007	Case-control study	Inhalation of toxic combustion products	The risk of developing leukemia did not differ significantly between firefighters and the non-exposed group (OR 1.22, 95% CI 0.99-1.49).
**4) Workers in petroleum processing facilities**				
Huebner [86]	2009	Cohort study	Byproducts of petroleum distillation and processing	A statistically significant increase of mortality from acute (non-lymphoblastic) leukemia was observed among workers in the chemical department (SMR 1.81, 95% CI 1.06-2.90), but no association with duration of employment or specific job posts was identified.
Gazdek [87]	2007	Ecological study	Emissions from oil and gas processing plants	Statistically significant geographic variation of acute myeloid leukemia incidence was observed among populations of different regions, depending on their proximity to oil- and gas-processing plants.
**5) Tannery workers**				
Iaia [88]	2006	Cohort study	Chemicals used in leather processing	No significant increase of myeloid leukemia mortality was found among tannery workers exposed to chemicals used for leather processing (SMR was 2.08, 90% CI 0.82–4.37 for men and 5.99, 90% CI 1.06-18.87 for women).
**6) Different occupational categories**				
Firth [89]	2007	Ecological study	Different occupational exposures of women	A significant increase of leukemia proportional mortality was found among women employed in the healthcare sector (PMR 1.52, 95% CI 1.08- 2.09) but not for nurses (PMR 1.42, 95% CI 0.96-2.01).
Hoffmann [90]	2008	Case-control study	Ionizing radiation, pesticides and EMF	15% of men (16% of women) participants reported occupational exposure to pesticides, 4% (8% women) reported exposure to ionizing radiation (for > 1 year) and 64% of participants reported having lived sometime in their life in the proximity (<20 km) of a nuclear plant.
Richardson [91]	2008	Case-control study	Different occupational exposures	A statistically significant increase of CLL risk was shown among workers exposed to polychlorinated biphenyls (PCBs) (OR 1.48, 95% CI 1.02-2.16) and printing inks (OR 1.89, 95% CI 1.21-2.96).
Kaufman [92]	2009	Case-control study	Benzene, pesticides, ionizing radiation and EMF	A statistically significant increase of myeloid leukemia was shown among workers exposed to Benzene (OR 3.9, 95% CI 1.3- 11), other non-specified solvents (OR 2.1, 95% CI 1.1- 4.9), pesticides (OR 3.8, 95% CI 2.1-7.1) and EMF (OR 4.3, 95% CI 1.3-15).
McLean [93]	2009	Case-control study	Different occupational exposures	A statistically significant increase of leukemia risk was found among workers employed in fruit and vegetable cultivation (OR 2.62, 95% CI 1.51 - 4.55) and in nurseries (OR 7.51, 95% CI 1.85-30.38), machine operators in plastic production facilities (OR 3.76, 95% CI 1.08-13.08), tailors and dressmakers (OR for CLL 7.01, 95% CI 1.78-27.68), cleaners (OR for CLL 2.04, 95% CI 1.00-4.14) and construction workers (OR for CLL 4.03, 95% CI 1.30-12.53).
**B) Occupational exposure and risk of leukemia among the offspring of different categories of workers**				
Pearce [94]	2006	Case-control study	Pesticides and herbicides	Paternal occupational exposure to pesticides and herbicides did not appear to be associated with a higher risk of leukemia among their offspring (OR 0.55, 95% CI 0.26-1.16 and 1.15, 95% CI 0.61 - 2.17 for children living in urban and rural areas respectively).
Pearce [95]	2007	Case-control study	EMF, ionizing radiation	A statistically significant association was shown between the risk of childhood leukemia and previous paternal occupational exposure to EMF (and ionizing radiation) (OR 1.31, 95% CI 1.02-1.69), especially among the offspring of electricians (OR 1.59, 95% CI 1.12 - 2.26).
McKinney [96]	2008	Case-control study	Solvents, degreasing and cleaning agents	A statistically significant association was found among acute lymphoblastic leukemia risk in children and maternal exposure to solvents, degreasing and cleaning agents, during the period of pregnancy (OR 2.7, 95% CI 1.6- 4.6) and postpartum (OR 1.9, 95% CI 1.1-3.3).
Perez-Saldivar [97]	2008	Case-control study	Carcinogenic compounds	A statistically significant association was found between childhood leukemia risk and previous paternal exposure to carcinogens (OR 2.06, 95% CI 1.24-3.42).

### Main findings

#### Ionizing radiation

As anticipated, the largest proportion of studies referred to ionizing radiation, which is to date the best documented risk factor for leukemia.

An update of Life Span Study findings has shown that exposure to ionizing radiation at doses as low as those usually recorded in occupational settings, leukemia incidence follows a quadratic dose response pattern, which peaks about 10 years following exposure and persists for decades [6,9]. Moreover, there is uncertainty on whether the proposed safety limits from the International Commission on Radiological Protection (ICRP) are appropriate, since revised LSS data show that the risk of leukemia remains increased even in groups with low cumulative exposure to radiation while most of the existing studies do not have sufficient statistical power to identify existing associations at such low exposure levels [98,99].

Ongoing observations on the impact of Chernobyl accident have shown that predictions based on LSS data present a high margin of error [100]. To date, a statistically significant increase of leukemia incidence has only been observed among the cleaning personnel that worked around the reactor site after the accident [29,30]. The study of Abramenko et al on specific genetic variants of CLL among this group of workers [28] implies a potential association among exposure to radiation and CLL which warrants further investigation given the lack of supporting evidence from previous studies.

As regards to a potential excess risk of leukemia among workers in the nuclear industry, the results of recent studies remain inconclusive. A number of studies among workers in nuclear-weapon industry have produced negative results [32-37] and despite sporadic positive findings, no significantly increased risk of leukemia has been established to date for nuclear power plant personnel [38,98,99] and other categories of workers involved in other industrial applications of ionizing radiation (medicine, research laboratories, non-destructive testing etc.) [39,40,43].

Exposure to minimal doses of radiation during the mining and processing of uranium has not been associated with a significantly increased risk of leukemia among exposed workers [45,46]. Moreover, despite existing weaknesses in their methodological design and statistical power [101], recent studies did not record any statistically significant increase in leukemia risk among military personnel exposed to depleted uranium products during military operations [44,102].

#### Non-ionizing radiation (EMF)

Occupational exposure to electromagnetic fields (EMF) constitutes an area of ongoing scientific debate over the recent years, mainly because of the rising public health concern regarding EMF. There has been high inconsistency among the results of previous studies, but the research group of the International Commission on Non-Ionizing Radiation Protection (ICNIRP) concluded that existing literature on EMF exposure converges on the existence of a small but statistically significant increase in leukemia risk among occupationally exposed groups [47]. These findings were further supported by more recent studies, which identified a small but statistically significant excess risk of leukemia among workers highly exposed to extremely low frequency electromagnetic fields (ELF EMF) [47,48].

#### Benzene

Among various chemical exposures in the workplace, Benzene is a well-documented risk factor for leukemia. As regards the potential toxicity of Benzene at cellular level, Zhang et al have observed in vitro a significantly higher number of genetic lesions associated with chemotherapy-related leukemia among Benzene-exposed workers [49], while Richardson et al have proposed a model of two-stage clonal expansion (TSCE) of cancer cells where Benzene acts as a promoter (rather than an initiator) affecting the kinetics of initiated blood cells [50]. A recent study in Great Britain estimated that due to the widespread use of benzene-containing compounds in various applications, occupational exposure to Benzene is responsible for about 0.19% of the overall incidence of non-lymphocytic leukemia in men and 0.34% in women [51], while a similar study in Korea has shown that Benzene constitutes at population-level a more significant occupational risk factor for leukemia than ionizing radiation [103].

#### Other organic solvents

Most epidemiological studies on occupational exposure to organic solvents other from Benzene have not investigated exposure to each compound separately, therefore the effect of co-exposure to benzene in the observed excess risk of leukemia could not be ruled out [52,53]. In a single study where exposure to organic solvents (Toluene, Hexane, Acetone, and Methyl-ethyl-ketone) was investigated independently from Benzene, no significantly increased risk of leukemia has been observed among the exposed workers [54].

#### Butadiene and dimethyl-dithio-carbamate

An interesting finding that warrants further research has occurred from a series of studies conducted on workers in the synthetic rubber industry, where a statistically significant excess risk of CML and CLL following a dose-response relationship was shown among workers exposed to 1.3-butadiene (BD) and dimethyl-dithio-carbamate (DMDTC) [57,59]. Interestingly, a similar finding was not observed among female workers, although their cumulative exposure to the specific compounds had been much smaller than men [60,61].

#### Formaldehyde

Although formaldehyde has been classified by the International Agency for Research on Cancer (IARC) in Group 1 “Carcinogenic to Humans” as regards to other types of malignancies [104,105], a potential role in the development of leukemia remains an area of ongoing scientific controversy, as epidemiological findings remain inconclusive [62,106] and no plausible theory has been proposed to date to explain a toxic effect on progenitors of blood cells [107]. In 2 recent Meta-analyzes of relevant studies however, a statistically significant excess risk of leukemia has been observed among those occupationally exposed to formaldehyde [108,109], while despite the criticism over its methodological weaknesses [110] an additional study has shown a statistically significant dose–response association between exposure to formaldehyde and mortality from myeloid leukemia among embalmers [63]. As regards the elucidation of the potential role of formaldehyde in the pathogenesis of leukemia, Zhang et al have reported in vitro evidence of formaldehyde myelotoxicity and identification of leukemia-characteristic genetic lesions among the myeloid lineage of formaldehyde-exposed workers [64], although there have been major objections to their findings by a different group of researchers [65]. Overall, while existing data could provide some explanation for the potential toxicity of Formaldehyde in remote target-organs and the negative findings in experimental animals, it seems that there is still a long way when it comes to document a role of Formaldehyde in the pathogenesis of leukemia [111].

#### Lead

The toxic effects of exposure to Lead on bone marrow have long been recognized. However, the cohort study of Lam et al. among lead-exposed workers (metal construction, metal processing, battery manufacture, glass production and electronics’ industry) has not detected any significant increase of leukemia risk [66].

#### Pesticides

Pesticides are a class of chemicals long suspected for carcinogenicity, and a number of different studies have been conducted over the last decade to investigate the toxic effect of specific classes of pesticides or compounds and their potential role in the development of leukemia. With the exception of an Italian case-control study which has shown no significant association between leukemia risk and exposure to the main categories of pesticides [69], a number of studies have recorded statistically significant excess risk of leukemia among agricultural workers exposed to pesticides in general [73,77], or specific categories of pesticides such as Phenoxy-herbicides, Triazine herbicides, Arsenic-containing pesticides, Atrazine, Lindane, Dichloro Diphenyl Trichloroethane (DDT), etc. [70,75]. A positive association was also shown between leukemia and exposure to organophosphate pesticide Fonofos [67], while for workers exposed to thio-carbamate herbicide EPTC [72], Organochlorine insecticides [71], Metribuzin [74] and Permethrin [76] no statistically significant excess leukemia risk was found.

#### Infectious agents – contact with animals

The potential association of leukemia with exposure to infectious agents is not a new theory but there seems to be a renewed interest as regards to the epidemiological investigation of this hypothesis during the last decade. A number of epidemiological studies have investigated the existing risk of leukemia among specific occupational groups in livestock farming, food production and processing where a higher theoretical risk of exposure to biological agents from the animal population exists [112-114]. The findings of those studies support the above hypothesis, as a statistically significant increase of leukemia risk was shown for workers in livestock farming [77,115], and a number of occupations i.e. workers in slaughterhouses, butchers, cooks, etc. involved in the processing of animal meat products [78-80].

#### Specific occupational groups at risk

Of the published epidemiological studies that investigated potential associations of leukemia with specific occupational groups over the last 5 years, a statistically significant increase in mortality from leukemia has been recorded among the personnel of chemical laboratories [84] and oil processing facilities [86], while no increased risk for leukemia was shown for firefighters [85] and tannery workers [88]. In 2 epidemiological studies conducted among healthcare personnel, no increased risk for leukemia was shown for the group of nurses [81] and neurosurgeons [83] while the findings of a third study which has shown increased mortality from CLL among nurses may be strongly affected by overdiagnosis of the disease due to the increased health awareness of the specific group [82]. Furthermore, a number of ecological-design studies have recorded a statistically significant excess risk of leukemia among specific categories of workers which include women (other than nurses) working in the healthcare sector [89], workers whose tasks involve exposure to Polychlorinated Biphenyls (PCBs) and printing inks [91], workers exposed to benzene or pesticides or working near high voltage lines [92], workers in plastic production, cleaning and construction work [93], and several categories of workers in the agricultural sector i.e. fruit and vegetable growers, nursery workers, farmers, florists etc. [93].

#### Parental occupational exposure and childhood leukemia

With regards to the potential association of childhood leukemia with parental occupational exposure, paternal exposure to pesticides and herbicides has not been associated with increased risk [94]. The study of Pearce et al has shown a potential association of paternal exposure to EMF with childhood leukemia, but their findings could be the effect of co-exposure to ionizing radiation [95]. Finally, paternal exposure to carcinogens [97] and maternal exposure to solvents [96] especially during pregnancy and after birth have been associated with increased risk of childhood leukemia among their offspring.

## Conclusions

The studies included in this literature review, present the current scientific knowledge on the potential effect of work-related hazards in the pathogenesis of leukemia. It must be noted, a number of those studies suffer from methodological weaknesses which in certain cases constitute their findings precarious.

Given the relatively low incidence and the long natural history of the disease, the investigation of a potential effect of an occupational exposure on leukemia requires large-scale cohort studies with long follow-up period to acquire the necessary statistical strength, which usually exceeds the resources or the size of the study cohort. Even when the above conditions are met, the statistical strength of a study can be significantly reduced if the study protocol for the assessment of exposure contains flaws. The establishment of the role of an occupational hazard in the development of leukemia assumes the demonstration of both a statistically significant impact on leukemia incidence or mortality between the exposed and non-exposed as well as a dose-response effect for different levels of exposure. In cases where available data on personal exposure is insufficient, as appears to be the case for a number of the cohort studies included in this review, an over- or under-estimation of exposure in certain groups of workers may have led to non-differential misclassification, reducing their statistical strength below the point where a weak (but existing) statistical association could be identified.

Case-control studies were susceptible to even more types of error, since apart from misclassification bias as regards the exposure (or the diagnosis, especially in studies based on historical records) major types of systematic error could undermine the reliability of their findings. Most case-control studies took place long after exposure, and the possibility of recall-bias, leading to an erroneous estimation of exposure, is high. Recall-bias could introduce a major error in the results of the studies especially when it is unevenly distributed among cases and controls (e.g. studies where exposure assessment was based on indirect information given by close relatives of colleagues of leukemia patients). Furthermore, some of the studies may have been affected by selection bias either as a result of a change in the composition of their reference population (e.g. migration of leukemia patients for diagnostic or therapeutic purposes outside a country may have led to over-representation of lower socio-economic classes and specific occupational groups among cases) or the over-representation of protective health behaviors among controls due to the followed sampling methodology.

A major concern with regards to published literature reviews and meta-analyses was the low comparability among the studies they were based on, as their different methodological design, analytical methods and presentation of results, made even simple comparisons among their findings difficult, let alone further processing of the data for the purpose of meta-analysis. This has affected some of the findings presented in this review, as some of the meta-analyses, as admitted by their own authors, could have reached completely different results if some of the large-scale studies which have been excluded from the analysis had met the criteria for inclusion.

Overall, during the reference period of this study there have not been any radical changes in the existing scientific knowledge as regards the role of work-related hazards in the pathogenesis of leukemia.

The findings of the reviewed articles confirm or fail to negate previous scientific observations on the effect of known risk factors, and there is adequate consistency among researchers as to the causal association between ionizing radiation or benzene and leukemia. Nevertheless, the results of different studies remain contradictory as regards the effect of radiation on different occupational groups, and the impact on leukemia risk at different exposure levels. Regarding exposure to benzene as a single compound, or as part of mixtures of solvents, the findings are consistent as to a positive association with the risk of leukemia among exposed workers, yet no agreement exists on its exact role on the development of the disease.

The epidemiological findings of different studies remain contradictory on the role of most of the remaining suggested risk factors. The positive associations shown by a number of studies as regards occupational exposure to EMF, BD or infectious agents warrant further research in the future, as different researchers fail to reproduce their statistically significant results. In the case of formaldehyde apart from the inconsistency of epidemiological findings as regards the effect on leukemia among exposed workers, there is also a scientific debate as to the plausibility of a theoretical model of action explaining its role in the development of the disease. Finally, the reviewed studies on occupational exposure to a number of plant protection products (pesticides, herbicides, insecticides) indicate a large heterogeneity among this group of chemical compounds as regards their potential effect on the risk of leukemia, and could provide an adequate explanation for the low reproducibility of findings among different researchers when characterization of exposure is not clearly defined.

The number of collected articles (especially cohort studies) investigating the potential association between different occupational hazards and leukemia, published during the previous years, indicates a growing scientific interest in the specific field in an attempt to improve our understanding on the causal factors of the disease. Nevertheless, based on the reviewed studies the potential effect of those hazards on the observed incidence and mortality of leukemia, if existing, is expected to be so subtle that could easily go unnoticed by small- or medium-scale epidemiological studies. Therefore, even negative results should be evaluated with caution, pending additional information from ongoing research.

Technology has provided new tools to researchers during the last decades for in vitro investigation of diseases like leukemia at cellular or molecular level. This will be perhaps the key to unraveling the pathophysiological mechanisms of this complex disease and acquire new insight on the actual causes of leukemia in the future, since epidemiology appears to have reached its limits as regards to introducing new knowledge in this field.

It is possible that, for diseases such as leukemia, the day when classical epidemiology will give its place to environmental genomics or proteomics to investigate complex interactions between occupational or environmental exposures and their effect at genetic or molecular level, is not far from today.

## Abbreviations

RR: Relative risk; ERR: Excess relative risk; Gy: Gray; LSS: Life span study; AML: Acute myeloid leukemia; CML: Chronic myelogenous leukemia; ALL: Acute lymphoblastic leukemia; CLL: Chronic lymphocytic leukemia; AL: Acute leukemia; PEL: Permissible exposure limit; ppm: Parts per million; TWA: Time weighted average; ICRP: International Commission on Radiological Protection; EMF: Electromagnetic fields; ELF EMF: Extremely low frequency electromagnetic fields; ICNIRP: International Commission on Non-Ionizing Radiation Protection; TSCE: Two-stage clonal expansion; BD: 1.3-butadiene; DMDTC: Dimethyl-dithio-carbamate; IARC: International Agency for Research on Cancer; DDT: Dichloro Diphenyl Trichloroethane; EPTC: S-ethyl-N,N-dipropylthiocarbamate; PCB: Polychlorinated Biphenyls; SIR: Standardized Incidence Ratio; OR: Odds Ratio; mSv: Millisievert; LAR: Life-time Attributable Risk; AF: Attributable fraction; APL: Acute Promyelocytic Leukemia; USAF: United States Air Force; TCDD: Tetrachloro-dibenzo-dioxin; CFU-GM: Colony Forming Unit-Granulocyte/Macrophage; AHS: Agricultural Health Study.

## Competing interests

The authors declare that they have no competing interests.

## Authors’ contributions

IP, GD, VM, ER, and AL have made substantial contributions to the conception and design of the review, acquisition of the review data and have been involved in drafting and revising the manuscript. All authors have read and approved the final manuscript.
